# Metabolic Maturation in hiPSC-Derived Cardiomyocytes: Emerging Strategies for Inducing the Adult Cardiac Phenotype

**DOI:** 10.3390/ph18081133

**Published:** 2025-07-29

**Authors:** Daniela Malan, Maria Pia Gallo, Federica Geddo, Renzo Levi, Giulia Querio

**Affiliations:** 1 Institute of Physiology I, Medical Faculty, University of Bonn, 53127 Bonn, Germany; dmalan@uni-bonn.de; 2 Department of Life Sciences and Systems Biology, University of Turin, Via Accademia Albertina 13, 10123 Turin, Italy; mariapia.gallo@unito.it (M.P.G.); federica.geddo@unito.it (F.G.); renzo.levi@unito.it (R.L.); 3 Department of Clinical and Biological Sciences, University of Turin, Regione Gonzole 10, 10043 Orbassano, Italy

**Keywords:** hiPSC-CMs, cardiomyocyte, metabolic maturation, metabolism, lactate, glucose oxidation, glycolysis, free fatty acid oxidation, mitochondria, oxidative metabolism

## Abstract

Human induced pluripotent stem cells (hiPSCs) are widely used in basic research because of their versatility and ability to differentiate into multiple cell types. In particular, differentiating hiPSCs into cardiac cells (hiPSC-CMs) has been an important milestone in cardiac pathophysiology studies. Although hiPSC-CMs offer a model for human cardiomyocytes, they still exhibit characteristics linked to the fetal cardiac cell phenotype. One important feature that prevents hiPSC-CMs from being identified as adult cells relates to their metabolism, which is a key factor in defining a mature phenotype capable of sustaining the workload requirements characteristic of fully differentiated cardiomyocytes. This review aims to present the most relevant strategies in terms of culture medium composition, culture times, and 3D culture methods that have been developed to promote the metabolic maturation of hiPSC-CMs, which are now widely used. Defining a standardized and universally accepted protocol would enable the creation of a cellular model for studies of cardiac pathophysiology from a patient-specific perspective and for drug screening.

## 1. Introduction

In the last decade, induced pluripotent stem cells (iPSCs) have become attractive models for studying development and disease mechanisms in vitro. The landmark report in 2006 by Shinya Yamanaka revolutionized biomedical research by identifying the four reprogramming “Yamanaka factors” Sox2, Oct3/4, Klf4, and proto-oncogene c-Myc [1]. Yamanaka remarkably created a new type of pluripotent cell termed iPSC from fully differentiated somatic cells [1] and earned the Nobel Prize for this discovery in 2012. Upon differentiation, iPSCs can generate all the somatic cell types and germ cells, thus fulfilling the criteria of pluripotency [2,3,4]. This discovery was an important breakthrough in stem cell research because, for the first time, it was possible to generate pluripotent stem cell lines that can be patient-specific and serve as disease models. Human iPSC (hiPSC) can generate an unlimited amount of syngeneic human tissue or cells, and the somatic cell origin reprogrammed by introducing the factors can overcome the ethical issues. Therefore, they may represent a more ideal source to produce patient-specific and disease-specific adult cells for future clinical applications and drug development.

A significant challenge for using human iPSC-derived cardiomyocytes (hiPSC-CMs) remains their functional maturation, which is crucial for enhancing the therapeutic potential in personalized medicine. It is known that cardiomyocytes (CMs) from fetal to post-natal undergo a process of metabolic maturation: at the fetal stage, they rely on glycolysis for energy production, whereas post-natal and mature CMs mainly utilize fatty acid oxidation. Moreover, metabolic maturation appears to be important for using hiPSC-CMs to gain insights into cardiac pathologies and diseases such as diabetes, which are based on alterations in metabolism. In this sense, the analysis of metabolic maturation processes gives information for understanding the onset of cardiac pathologies.

Studies have shown that hiPSC-CMs are metabolically similar to early neonatal hearts, relying primarily on glycolysis and lactate oxidation to produce ATP and very little on fatty acid oxidation [5]. The metabolic immaturity of hiPSC-CMs limits their ability to replicate adult-like contractile properties; notably, mature cardiomyocytes rely on a well-developed sarcomere structure and efficient ATP generation to sustain forceful contractions. Therefore, it is essential to achieve a transition of hiPSC-CMs towards a metabolism that favors fatty acids to improve their functional integration in disease modeling and efficacy in cell-based regenerative therapies. Efforts to enhance their maturity through biophysical stimulation, substrate stiffness modulation, combined with 3D organotypic cultures that enhance metabolic activity and oxygen consumption rates, and metabolic conditioning are ongoing but have yet to fully bridge the gap between hiPSC-CMs and adult cardiomyocytes [6,7,8]. This review aims to explore the mechanisms governing metabolic maturation in hiPSC-CMs, discuss current strategies to enhance maturation, and highlight the implications of these advancements for studying metabolic disorders-related cardiac diseases and drug screening.

## 2. Cardiac Metabolic Maturation

Metabolic maturation of cardiac cells is the process by which cardiomyocytes adapt to produce energy efficiently and sustainably. This process is vital for the development of a fully functional heart. The opportunities for patient-specific disease modeling and drug development are limited by the metabolic maturation state of these cells, which remains a principal obstacle to their full translational use.

### From Fetal to Adult Heart: Mitochondria and Metabolic Switch

Mitochondria are important regulators of cellular metabolism. An interesting review by Persad and Lopaschuk [9] comprehensively describes the close relationship between mitochondria and cell fate.

As with other cell types, for cardiomyocytes, the regulation of mitochondrial fission, fusion, mitophagy, and biogenesis is crucial for their maturation and the determination of the metabolic fate of the cell. In particular, mitochondrial fission, that is, the separation of one mitochondrion into two, turns out to be very important in the cell proliferation stages, such as for cardiomyocytes during the fetal and neonatal periods of development. In the postnatal stages, with the progression of cell differentiation, there is a reduction in mitochondrial fission and an increase in mitochondrial fusion, that is, the union of two mitochondria into one, thus characterizing mitochondrial maturation.

Mitophagy, i.e., the regulated degradation of mitochondria, plays an important role in cardiac cell maturation: in fact, it reduces the number of damaged mitochondria, thus selecting the energy-efficient ones and promoting metabolic maturation. This process is closely related to mitochondrial biogenesis, which allows the maintenance and growth of high-quality organelles.

In addition, mitochondria change morphology and location during cardiac metabolic maturation, underscoring the importance of these organelles in cellular metabolic regulation. In particular, their size and association with sarcomeres are indicative of their mutual influences in the maturation process. Mitochondria of immature cells have a round shape, and cristae do not yet appear as dense as those of mitochondria of adult cardiomyocytes [9,10]. Furthermore, mitochondrial morphology is strongly linked to the energy requirements of the cell: the not-yet-complex organization of the fetal and perinatal stages is related to the prevalence of glycolytic metabolism to provide the proper ATP supply to the developing cell [9].

Interestingly, during the maturation of hiPSC-CMs, mitochondria and sarcomeres are often interconnected and reciprocally regulate their maturation. Improved mitochondrial structure, mass, and oxidative function usually coincide with highly organized sarcomeres and the upregulation of contractile proteins such as MYH7 and TNNI3 [11,12]. However, some studies have shown that mechanical or electrical stimulation may promote mitochondrial maturation and bioenergetic capacity without inducing consistent changes in sarcomere isoform expression or structural organization, and vice versa [12,13,14,15,16]. Furthermore, deleting sarcomeric genes in mice, such as *MYH6* and *ACTN2*, disrupts the formation of sarcomeres and T-tubules, leading to altered mitochondrial organization. Nevertheless, mitochondrial content remains intact, suggesting partial independence between the two components [17]. Furthermore, serum response factor knockdown has been shown to result in more pronounced defects in sarcomere organization than in mitochondrial biogenesis. As suggested by Guo et al., the regulation of cellular maturation in cardiomyocytes follows a hierarchy with sarcomere assembly at the beginning. Sarcomeric maturation appears to be essential for subsequent mitochondrial biogenesis and morphological maturation. However, altering mitochondrial dynamics alone is insufficient to replicate the full loss of the maturation phenotype [18]. In hiPSC-CMs, mitochondrial maturation induced by substrate manipulation, such as triiodothyronine (T3) supplementation, does not always result in parallel changes in sarcomere organization [19]. Switching gene isoforms from *MYH6* to *MYH7* or *TNNI1* to *TNNI3* does not always correlate with maturation levels, despite metabolic maturation [16,19].

In light of the changes that characterize the main regulators of cellular energy, it is necessary to describe the metabolic evolution in cardiomyocytes from fetal development to adult maturation. In fact, in the early stages of fetal development, cardiac cells use glucose as the main energy substrate and, through the process of glycolysis, support the production of ATP required for cellular energy and anabolic demands.

In the postnatal stages, cardiomyocytes are characterized by a metabolic switch that progressively results in a diminishing role of glycolysis and a predominance of oxidative phosphorylation. A unique feature of adult cardiomyocytes is that the main substrates of oxidative phosphorylation are free fatty acids (FFAs) rather than glucose, covering up to 70% of the cellular requirement in the adult phase. A study by Lopaschuk and coworkers highlighted this substantial change in the metabolic substrates in the newborn rabbit hearts [20]. The authors showed that the 1-day postpartum heart obtains ATP mainly from glycolysis (44%), while palmitate and lactate oxidation provide 13% and 25% of ATP, respectively. The study also showed that hearts from 7-day-old rabbits displayed reduced glycolytic processes (7% of total ATP) and increased oxidation of FFA and lactate, which provide 39% and 49% of total ATP, respectively [20]. The same authors showed that in hearts at 21 days after birth, FFAs constitute the main source of ATP, reaching a supply of about 80% [21]. According to the authors, this is due to the increased availability of FFA as the animal ages and the increased expression of enzymes involved in lipid oxidation processes [20].

Persad and Lopaschuk [9] describe the Warburg effect in fetal cardiomyocytes, which is a metabolic behavior that primarily characterizes tumor cells. In other words, even in the presence of oxygen, these cells continue to perform glycolysis, resulting in increased lactate production. During the postnatal period, the Warburg effect decreases progressively, and oxidative phosphorylation increases [9].

Moreover, this metabolic switch that characterizes the adult phase of cardiomyocytes is favored by a higher availability of substrates and the greater amount of ATP produced from fatty acid oxidation than from glucose oxidation [22].

A comprehensive review by Bornstein et al. describes the difficulty in collecting human samples to study cardiac metabolism [23]. Indeed, cardiac tissue sampling is an invasive and not-easy-to-perform but valuable method to study ex vivo human samples. The authors illustrate other non-invasive techniques that can be used to compare cardiac metabolic substrates in health and disease, such as arterio-venous sampling, 2-deoxy-2-[^18^F]fluoro-D-glucose (^18^FDG) for positron emission tomography (PET) scanning, 4-compartment model of ^11^C-palmitate uptake and oxidation, and phosphorus magnetic resonance spectroscopy (^31^P-MRS). In particular, the authors focus on the bioavailability of the different substrates, but also on the ability of cardiac cells to use them [23]. Specifically, in a healthy individual, cardiac energy supply is mainly represented by FFA, and, to a lesser extent, by glucose and lactate, and only in small percentages, ketone bodies and amino acids were recorded. In patients with heart failure with reduced ejection fraction, glucose and lactate uptake decline, resulting in FFA as the major energy fuel component. This diversity of metabolic substrates used by cardiac tissue is representative of what is termed the metabolic flexibility of the adult heart in response to different environmental stimuli.

The metabolic switch that characterizes cardiomyocytes from the fetal to the adult stage is also distinguished by a change in membrane-expressed transporters. Specifically, FFA can be transported intracellularly either by passive diffusion or through membrane transporters. The main ones identified are fatty acid translocase (FAT)/CD36, plasma membrane fatty acid-binding protein (FABPpm), and fatty acid transport protein (FATP) 1/6. FAT/CD36 has been shown to play a predominant role in FFA uptake by adult cardiomyocytes, and reduced expression of FAT/CD36 has been linked to impaired FFA metabolism in the heart [24].

**Figure 1:**
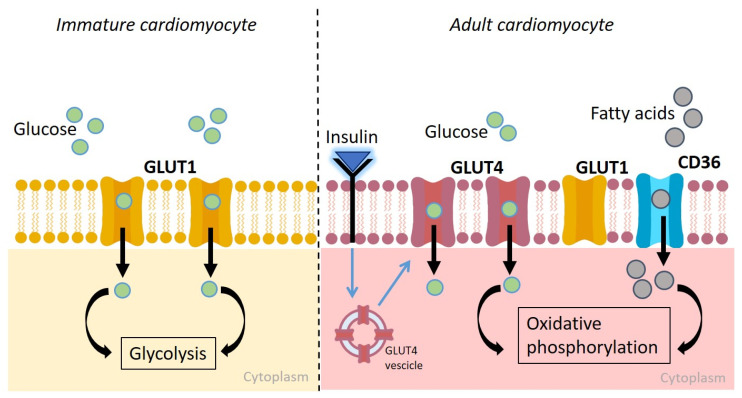
Schematic representation of cardiomyocyte metabolic maturation. In immature cardiomyocytes, GLUT1 (yellow) is the major glucose transporter in the plasma membrane, and glycolysis is the main metabolic pathway. In adult cardiomyocytes, the oxidative phosphorylation of fatty acids is the primary energy source, and CD36 (light blue) mediates fatty acid uptake. The uptake of glucose in adult cardiomyocytes is mediated by the insulin-dependent transporter GLUT4 (purple). Image created with Canva.com.

Instead, glucose uptake is mediated by several transporters that undergo an expression switch from the fetal to the adult stage. During the early stages of fetal development, in which the glycolytic process prevails, the main glucose transporter expressed in the membrane is GLUT1. With metabolic maturation, characterized by a reduction in glycolysis, there is a progressive reduction in GLUT1 transporters and an increase in the expression of the insulin-dependent GLUT4 transporters, becoming the most abundant isoform in the adult cardiomyocytes [25,26] (Figure 1). Recently, GLUT1 and GLUT4 transporter regulation and expression were investigated in hiPSC-CMs, leading to controversial results, probably due to the different cellular origin of the tested hiPSC-CMs. Indeed, in one experimental model, GLUT1 appeared to be highly expressed, while GLUT4 levels were significantly lower and did not respond to insulin stimulation. Thus, the authors concluded that hiPSC-CMs are not a reliable model for studying insulin-dependent glucose metabolism or diabetic cardiomyopathy due to their immature, fetal-like metabolic phenotype [27]. On the contrary, another study found that hiPSC-CMs can translocate GLUT4 to the plasma membrane in response to insulin, suggesting they are metabolically relevant [28]. The peculiarity of this second study was based on the physiological mechanism that regulates GLUT4 membrane expression. Indeed, the authors, rather than focusing on total cell lysates, analyzed membrane fractions, revealing that insulin activates AKT and AS160 phosphorylation, the principal drivers of GLUT4 membrane translocation [28]. Moreover, the latter study used lactate medium to selectively isolate cardiomyocytes, potentially promoting a more mature phenotype and influencing GLUT4 expression and translocation. This suggests that analytical techniques and culture conditions can significantly impact the metabolic maturity of hiPSC-CMs.

Interestingly, it is now known that disease states trigger a reversion of metabolism, with a shift back to glycolytic processes, also accompanied by re-expression of GLUT1 transporters [21,24,29]. Therefore, developing a metabolically accurate cellular model that mirrors these and other pathological changes represents a critical research goal.

## 3. Strategies to Induce Metabolic Maturation in hiPSC-Derived Cardiomyocytes

As previously discussed, studying isolated human heart tissue is complicated by the invasiveness of sample collection and the reduced viability of cardiac cells once isolated and cultured [30]. To overcome these ethical problems associated with the study of patient-derived cardiac cells, the use of hiPSC-CMs has become a well-established model. The advantages of hiPSC-derived cells are linked to their human origin, the non-invasive techniques for their generation, and the large number of cells that can be produced.

Differentiation of hiPSC-CMs is a widely used technique, and although the protocols differ in some respects, they generally involve sequential steps such as initial differentiation into cells of the embryonic mesoderm layer, specification of cardiac progenitors, cardiac differentiation, and finally maintenance and maturation of cardiomyocytes [31]. However, many studies show that hiPSC-CMs do not recapitulate adult cardiomyocyte characteristics and that they are much less mature [32,33] from a morphological, electrophysiological, and metabolic point of view.

In particular, as anticipated in the introductory chapter, the importance of having metabolically mature hiPSC-CMs is an undisputed advantage for the study of metabolic diseases, such as diabetic cardiomyopathy. Although protocols for differentiating hiPSC-CMs are now standardized, there is still no common understanding of the best approach to promote metabolic maturation that brings these cells as close as possible to adult cardiomyocytes.

The following sections, summarized in Table 1, illustrate the most widely used techniques in the literature for the induction of metabolic maturation in hiPSC-CMs.

**Table 1:** Different and most used techniques to induce metabolic maturation of hiPSC-CMs.

Maturation Technique	Effect	Reference
Long-term culture	After 100 days of culture, mitochondrial relative abundance, mitochondrial membrane potential, and activity of respiratory complexes I–III increased.	[34]
	200 days of culture result in metabolic maturation by promoting the shift from glycolysis to β-oxidation and activation of the cAMP-PKA-proteasome axis.	[35]
	Stimulation with isoproterenol (100 nM) led to a greater increase in the extracellular acidification rate at day 45 than at day 37.	[36]
	After 12 weeks of post-differentiation culture, hiPSC-CMs showed increased ability to oxidize fatty acids and reduced glucose oxidation. Structural and functional maturation of mitochondria.	[37]
Hormonal regulators	Triiodothyronine (T3) treatment for 1 week after differentiation induces an increase in respiratory reserve capacity.	[38]
	Treatment with triiodothyronine, insulin-like growth factor-1, and dexamethasone (TID) between days 16 and 21 of differentiation resulted in increased respiratory rate.	[39]
	TID treatment increased expression of long-chain fatty acid transporter CD36, mitochondrial uncoupling protein UCP3, fatty acid synthase, GLUT4, and pyruvate dehydrogenase kinase 4.	[40]
Metabolic substrates	Lactate-containing medium induced higher GLUT4 membrane translocation after insulin stimulation.	[28]
	Cultivation for 3–5 weeks in a maturation medium containing a complex mixture of albumin-bound fatty acids induced a metabolic maturation with higher levels of fatty acid oxidation.	[11,41,42]
	Maintenance medium for 20 days, composed of galactose, oleic acid, and palmitic acid, improves the oxidative capacity of hiPSC-CMs.	[43]
	Incubation with lactate medium for the first 7 days after differentiation and a subsequent incubation for 3 to 7 days in glucose-free medium enriched with linoleic acid induced an adult-like metabolic profile.	[44]
	Galactose rather than glucose in the medium improves metabolic maturation.	[45]
Functional maturation	Electrical conditioning to promote mitochondrial maturation and increase oxidative metabolism in engineered cardiac tissues does not show consistent maturation in sarcomere isoform expression.	[12]
	Electrostimulation promotes structural and electrophysiological maturation with benefits on action potential propagation and contraction synchronization, increased mitochondrial content, oxidative capacity, and more developed sarcomere structure.	[46]
Single-culture and multi-culture cardiac 3D models	Single-culture of 3D hiPSC-CMs shows downregulation of glycolytic genes and upregulation of mitochondrial oxidative phosphorylation genes matched by an increase in fatty acids oxidation and a reduction in glycolysis.	[47,48]
	Functional increase in oxidative metabolism but equal rates of glycolysis between undifferentiated and differentiated hiPSC-CMs spheroids.	[49]
	3D multicellular cultures show elongated mitochondria and express genes involved in fatty acid β-oxidation.	[7,50]
	3D multicellular cultures and crosstalk between cardiomyocytes, endothelial cells, and fibroblasts are essential for the metabolic maturation of hiPSC-CMs.	[7]
	Engineered heart tissue (EHT) as a 3D model shows fatty acid oxidation activation and modulation with oleate. Stimulation of peroxisome proliferator-activated receptor alpha (PPARα) in 3D EHT further increased oxidative metabolism.	[51]
	Long-term human organotypic cardiac microtissues (hOCMs), scaffold-free multicellular beating human cardiac microtissues, show elongated mitochondria, express genes involved in fatty acid β-oxidation, and rely more on mitochondrial respiration.	[50]

### 3.1. Long-Term Culture

Several studies have shown that prolonging culture time in hiPSC-CMs results in structural and mitochondrial maturation, changes which can be attributed to metabolic maturation.

Dai and coworkers demonstrated that hiPSC-CMs kept for 100 days in culture exhibited enhanced mitochondrial biogenesis, increased mitochondrial relative abundance, enhanced mitochondrial membrane potential, and increased activity of respiratory Complexes I–III [34]. However, the authors conclude that prolonged culture times were not sufficient to achieve mitochondrial maturation comparable to that which occurs in vivo.

In addition, Ebert and coworkers have shown that hiPSC-CMs cultured for 30, 90, and 200 days exhibit maturation very similar to that characterizing the physiological maturation of cardiomyocytes [35]. Indeed, the authors showed that cells cultured for 30 days exhibited a metabolism still dependent on glycolytic processes, while cells cultured for longer periods showed a metabolic shift towards β-oxidation. Furthermore, longer culture times are associated with metabolic regulation by the cAMP-PKA–proteasome axis, whose activation is found to be higher in hiPSC-CMs after 200 days of culture.

Conversely, stimulation of hiPSC-CMs with isoproterenol (100 nM) led to a greater increase in the extracellular acidification rate, an indicator of glycolysis, at day 45 rather than at day 37 [36].

Emanuelli et al. showed metabolic remodeling in hiPSC-CMs cultured for 12 weeks [37]. Specifically, they showed that the ability to oxidize fatty acids increased over time, in parallel with a reduction in glucose oxidation. These changes in metabolic substrate utilization were accompanied by the structural and functional maturation of the mitochondrial network. In addition, prolonged culture times resulted in increased organization and regularity of the sarcomeres.

### 3.2. Hormonal Regulators

Given the important role that hormones such as T3, glucocorticoids, and insulin-like growth factor-1 (IGF-1) play in cardiac development in vivo [52,53,54], several studies have evaluated their role and impact on the metabolic maturation of hiPSC-CMs.

Yang and coworkers demonstrated that treatment with T3 for 1 week after hiPSC-CMs differentiation induced a more mature metabolic phenotype and increased respiratory reserve capacity. The authors associated these changes with an improved performance under increased energy demands [38]. Birket et al. [39] evaluated the combined role of T3, IGF-1, and dexamethasone (TID) treatment from day 16 to day 21 of hiPSC-CMs differentiation. A higher respiratory rate was shown after differentiation, suggesting the utilization of both pyruvate and FFA as energy substrates. Moreover, in a study conducted on 3D cardiac spheres, an increase in the oxidation of FFA and other substrates was shown following TID treatment. There was also evidence of a 4.8-fold increase in the long-chain fatty acid transporter CD36, a 2.2-fold increase in the mitochondrial uncoupling protein UCP3, and a 3.1-fold increase in the fatty acid synthase (FASN), as well as an increase in the expression of GLUT4, the glucose transporter isoform most highly expressed in adult cardiomyocytes. The authors also showed a 2.7-fold increase in pyruvate dehydrogenase kinase 4 (PDK4), a key enzyme involved in the regulation of glucose and FFA metabolism and the inhibition of glucose oxidation [40].

### 3.3. Metabolic Substrates

As mentioned in the introduction, cardiomyocytes undergo a metabolic switch during their maturation. Therefore, it is reasonable to induce the metabolic maturation of hiPSC-CMs by modulating the energy substrates in culture media.

Using a lactate-containing medium rather than glucose as the sole energy substrate yielded good results in terms of cellular purification, given the ability of cardiomyocytes to use lactate to produce energy to support all physiological functions [55]. Indeed, Kadari et al. showed that incubating hiPSC-CMs in a lactate medium after differentiation increases the relative percentage of troponin T-positive cells from 63% to 95% [56].

From a metabolic point of view, hiPSC-CMs cultured in lactate medium for three days after differentiation showed GLUT4 membrane translocation due to pAKT and pAS160 activation after insulin stimulation [28], as occurs in adult healthy cardiomyocytes [26].

Feyen and collaborators demonstrated that cultivation of hiPSC-CMs for three to five weeks in a maturation medium containing low levels of glucose and a complex mixture of albumin-bound fatty acids induced a metabolic maturation, characterized by higher levels of fatty acid oxidation [11].

Moreover, Correia et al. conducted an interesting study that examined the impact of different maturation media on metabolic changes in hiPSC-CMs. In particular, the authors highlighted that incubating the cells in a medium containing 43% galactose, 8% oleic acid and 11% palmitic acid for the first 10 days after differentiation, followed by 10 days in a medium containing 46% galactose, 19% oleic acid, and 17% palmitic acid, improves the cells’ oxidative capacity without lipotoxicity [43]. Similar results were obtained after incubation for 30 days of hiPSC-CMs with FFA, which increased oxygen consumption rate and the use of fatty acids as the primary energy source [41], while incubation for 20 days with a medium enriched with FFA increased the expression of cardiac maturation markers [42].

Furthermore, Horikoshi and collaborators suggested a combined effect of lactate medium for the first seven days of differentiation, followed by an incubation period of three to seven days in a glucose-free medium enriched with linoleic acid [44]. At the end of these differential treatments, the authors observed an adult CM-like metabolic phenotype, characterized by improved mitochondrial oxidative function, the use of fatty acids as energy substrates, as well as the ability to switch to glucose as an energy source when needed [44].

Finally, a recent work by Fetterman and collaborators found that, in the same medium composition, galactose rather than glucose improves the metabolic maturation of hiPSC-CMs [45]. These presented and other studies demonstrate the importance of single components of hiPSC-CMs medium for their metabolic maturation.

### 3.4. Functional Maturation

In addition to modulation of the metabolic substrate, functional signals, particularly those related to contractile activity and conduction propagation of the electrical signal, may influence the metabolic maturation of hiPSC-CMs.

The workload of the contractile function of CMs increases during development; therefore, electrical conditioning of CMs has been used in several studies as a functional manipulation to promote mitochondrial maturation and increase oxidative metabolism in engineered cardiac tissues, sometimes without consistent maturation in sarcomere isoform expression [12]. In a recent study by Li and coworkers, electrostimulation was proven to promote structural and electrophysiological maturation, with benefits including improved action potential propagation and more synchronized contractions, leading to increased mitochondrial content, oxidative capacity, and a more developed sarcomere structure [46]. This study showed that integrating fatty acid substrate modulation, electrophysiological properties, and nanopatterned substrate-forcing alignment conditioning produced the most robust maturation phenotype. This strategy induced the most comprehensive adult-like phenotype, including action potential characteristics such as a more negative resting membrane potential, a higher amplitude, a faster upstroke velocity due to mature Na^+^ channels, a distinctive plateau phase, and notch-and-dome morphology. Additionally, conduction velocity nearly doubled (from 12.5 cm/s to 27.8 cm/s), approaching values found in adult myocardial tissue. Contractile function also strengthened and became faster.

Together with electrical pacing, the mechanical loading and stretching of engineered heart tissues can improve contractile properties and metabolic signatures like increased oxygen consumption rate and fatty acid oxidation. Moreover, 3D constructs integrating mechanical and electrical stimuli with cell–cell and cell–matrix substrates further accelerate the metabolic switch to more mature states [11,46,57,58] (see also the paragraph which follows). The impact of such models is related to a higher physiological relevance; however, it is still needed to optimize protocols for scalability and reproducibility [46].

### 3.5. Single-Culture and Multi-Culture Cardiac 3D Models

The lack of tissue-specific architecture, including cell–cell and extracellular matrix (ECM)–cell interactions, appropriate morphology, and electrophysiological as well as structural maturation, shows that hiPSC-CMs 2D cultures do not accurately represent the real cellular environment. To address these limitations, three-dimensional (3D) culture systems rather than monolayer culture of hiPSC-CMs have emerged as superior models for studying both physiological and pathological cardiac conditions. Among these systems, spheroids and organoids are particularly promising, as they better mimic the structural, functional, and microenvironmental complexity of in vivo tissues.

Three-dimensional culture, like spheroids, generated simply from hiPSCs assembly, shows that differentiated hiPSC-CMs exhibit different metabolic patterns compared to 2D cultures [59]. In particular, 3D models display the downregulation of glycolytic genes and upregulation of mitochondrial oxidative phosphorylation genes, thus highlighting a more mature metabolic profile [47]. These changes in 3D cultures are also observed at the transcriptomic level, where this difference becomes more pronounced with longer culture times [47]. These results were supported by Ulmer and collaborators, who demonstrated an increase in fatty acid oxidation and a reduction in glycolysis in 3D hiPSC-CMs compared with 2D models [48]. However, these results are in contrast with those by Bobori et al., who reported a functional increase in oxidative metabolism but equal rates of glycolysis between undifferentiated and differentiated hiPSC-CMs spheroids [49]. This discrepancy may be due to variations in spheroid size, culture media, and differentiation protocols.

Organoids are more complex structures derived from stem or progenitor cells, capable of self-organizing in a complex model similar to a fully functional organ, thus enabling investigations of organ development, genetic diseases, and personalized medicine. Recent research highlights the critical role of microenvironmental factors, such as different cell types (i.e., endothelial cells and fibroblasts in the heart), the extracellular matrix, and dynamic flow in organoid generation. Indeed, Giacomelli and collaborators highlighted that in 3D multicellular cultures, crosstalk between cardiomyocytes, endothelial cells, and fibroblasts is essential for the metabolic maturation of hiPSC-CMs [7]. Moreover, 3D extracellular matrices such as fibrin, collagen I, and Matrigel have been used to mimic the organ microenvironment to induce differentiation and maturation [6]. In a study using engineered heart tissue (EHT) as a 3D model, fatty acid oxidation was activated by modulating the culture medium with oleate. Stimulation of peroxisome proliferator-activated receptor alpha (PPARα) in 3D EHT further increased oxidative metabolism [51]. Several other scaffold-free methods have been developed to generate organoids. In the cardioid model described by Hofbauer [60], the intrinsic self-assembly of hiPSCs and their differentiation into CMs, endocardial, and epicardial cells, together with secreted ECM, stimulates the Wnt-BNP axis and HAND1 transcription factor, leading to tissue patterning and cavity-containing structures resembling early heart chambers. Lewis-Israeli et al. [61] developed a scaffold-free, self-organizing human heart organoid model that displays internal chambers, sarcomere organization, and vascular-like structures. They also observed both atrial and ventricular cardiomyocyte populations in specific regions of the 3D structures, thus mimicking human heart tissue organization. Furthermore, long-term human organotypic cardiac microtissues (hOCMs)–scaffold-free multicellular beating human cardiac microtissues in vitro exhibit features of metabolic maturity. Compared to 2D models, hOCMs show elongated mitochondria, express genes involved in fatty acid β-oxidation, and rely more on mitochondrial respiration. These features correlate with improved contractile force and electrophysiological stability, thus underlining a more mature phenotype [50].

In recent years, significant efforts have been directed towards developing organoids with enhanced cellular maturation, particularly for cardiomyocytes. Most of these models rely on scaffolds that use hydrogels or matrices to support 3D structure formation. Chamber-specific organization—towards atrial or ventricular identity—was achieved using an exogenous scaffold hydrogel combined with modulation of biochemical cues, such as retinoic acid gradients [62]. Alongside coculture conditions, which still need a more effective integration technique, the supplementation of nutrients and oxygen for long-term fluid flow cultures has been analyzed [8]. Using a decellularized heart ECM hydrogel, a dynamic perfusion was achieved via a microfluidic chip. The advantage of this study lies in its in vivo-like ECM microenvironment and microfluidic perfusion system, enabling the long-term maturation and upscaling of organoids up to 1.2 mm.

The new generation of scaffold-based 3D cultures offers better reproducibility and control over size and architecture compared to self-assembling organoids. The scaffold’s mechanical stability enables patterning, biochemical cue integration, and fluid perfusion. However, scaffold-based organoids face challenges: they are more expensive, require specialized techniques, and are harder to standardize than 2D systems. While 3D organoid systems offer enhanced complexity, they are limited in analysis methods. A recent report addresses this issue by integrating stretchable, tissue-embedded nanoelectronics, which enable long-term high-resolution electrophysiological recordings. Machine learning analysis of this data confirmed that endothelial cells promote cardiac maturation by upregulating functional genes and ECM [63]. Despite various advances, none of these models has yet succeeded in recreating a fully adult cardiac model, either metabolically or functionally.

## 4. HiPSC-CMs as a Tool for Disease Modeling and Drug Screening

### 4.1. Three-Dimensional Cultures to Model Metabolic Cardiac Diseases

Examples of cardiovascular pathologies shaped on 3D structures include the organoid model developed by Lewis-Israeli et al. [61] to study pregestational diabetes-induced cardiomyopathy, as it recapitulates features of heart development. The disease state can be replicated by modulating the metabolic profile to affect glycolysis and mitochondrial respiration. Adjustment of the media to mimic hyperglycemic and hyperinsulinemic environments resulted in a larger size of human heart organoids, mimicking fetal overgrowth in diabetic pregnancies. Additionally, cardiomyocyte subpopulations shifted towards higher atrial expression with fewer mitochondria and increased glycolysis, matching the metabolic dysfunction associated with oxidative stress and lipotoxicity. Furthermore, the researchers investigated the signaling pathway linking maternal diabetes to fetal cardiomyopathy, observing enhanced markers of endoplasmic reticulum stress (IRE1-RIDD pathway) with inhibition of very long-chain fatty acids. By identifying very long-chain fatty acids as critical mediators of cardiac development and function, they were able to explore potential therapeutic targets such as IRE1 inhibitors or VLCFA supplementation. Other self-organizing organoids can recapitulate epicardial mesenchymal transition and myocardial compaction, and have been used to model congenital heart disease, stress-induced cardiac hypertrophy, and fibrosis [64,65], as well as to study early development. Moreover, the combination of an exogenous scaffold hydrogel combined with biochemical cue modulation, such as retinoic acid gradients, has successfully recapitulated congenital defects such as Ebstein’s anomaly using NKX2.5 mutant-derived chamber organoids [62].

Thus, scaffold-free organoids enable the study of endogenous development and disease processes, such as pregestational cardiomyopathy, through self-generated ECM–cell interaction, which is more similar to the in vivo situation. However, they may lack the biomechanical forces of scaffold-supported organoids and be subject to size limitations. These systems are relatively low-cost, require minimal technical expertise, and have proven suitable for drug validation.

A vascular organoid model, embedding hiPSC-derived spheroids in a 3D scaffold matrix of collagen I and Matrigel, demonstrated vascular network sprouting and maturation. This system effectively modeled diabetic vasculopathy: when generated from hiPSCs of diabetic donors, the vascular organoids exhibited impaired vascular function, including dysregulated signaling networks, proinflammatory cytokine elevation, increased reactive oxygen species, and reduced angiogenesis. The authors identified upregulated GAP43 expression in endothelial cells from diabetic vascular organoids, suggesting a novel mechanism linked to vascular development and diabetic vasculopathy, which could potentially be targeted therapeutically [66].

### 4.2. HiPSC-CMs in Drug Screening Models

Undoubtedly, one of the most innovative applications of hiPSC-CMs is their use in preclinical drug screening studies. As previously mentioned, hiPSC-CMs have shown morphological, phenotypical, and functional similarities with adult cardiomyocytes. This enabled their use in the study of cardiovascular diseases, the identification of promising drug candidates in high-throughput screening, the development of patient-specific therapy, as well as cardiotoxicity assessment. Responses to drugs by hiPSC-CMs have been shown to have a very similar profile to those of native counterparts. They are, therefore, emerging as a possible bridge between preclinical and clinical studies [67]. Some pathological conditions modeled on hiPSC-CMs, including long QT (LQT) syndromes [42,68,69], torsade de pointes and other arrhythmias [59,70], have shown similar pharmacological responses to the patient counterparts. As an example, mexiletine and ranolazine shorten the durations of action and field potentials specifically in LQT3 hiPSC-CMs, and antagonize early after depolarizations in a dose-dependent manner, demonstrating the beneficial effects of its administration in LQT3 patients and the reliability of hiPSC-CMs for drug screening [69]. Also, a recent study from Crotti indicates the use of hiPSC-CMs to investigate the therapeutic efficacy of mexiletine for long QT Syndrome Type 2, comparing these findings with both the animal rabbit LQT2 model and human patients [71]. Some initiatives, such as the Comprehensive in vitro Proarrhythmias Assay (CiPA), are trying to standardize the use of hiPSC-CMs to predict pro-arrhythmic risk in drug discovery, and both the study from the group of Blinova [72] and the group of Kanda [73] demonstrated that hiPSC-CMs offer a platform for preclinical cardiac safety screening, categorizing more than 28 drugs with low (mexiletine), intermediate (quinidine), and high (dofetilide) pro-arrhythmic risk. Some studies support the idea that hiPSC-CMs reduce false positives compared to traditional hERG assays, as, for instance, verapamil was correctly identified as a low-risk drug [74].

Kussauer and collaborators have reviewed in great detail all the studies in which hiPSC-CMs have been used to study the effect of drugs and substances commonly administered to counter non-cardiac diseases, but which have demonstrated side effects precisely in terms of cardiotoxicity [75]. For instance, cardiotoxicity induced by doxorubicin, an anthracycline used to treat a wide range of tumors, has been tested on different patient-derived hiPSC-CMs, underlining the important role of utilizing patient-derived cells to identify specific cardiac side effects and distinguish different degrees of cardiac damage related to genetic background [76,77,78].

It is curious that in this very detailed overview of all cardiac disease modeling studies and cardiotoxic drug testing, metabolic diseases that have detrimental cardiac implications, such as diabetic cardiomyopathy, are not mentioned. This is probably because the full metabolic and functional characteristics of diseased cardiomyocytes are not yet fully understood [79]. The lack of studies on dysfunctions in cardiac metabolism is a major shortcoming from the perspective of personalized and patient-specific medicine.

Enormous strides have been made through the use of hiPSC-CMs in both cardiac disease modeling and pharmacological studies; however, it is important to emphasize that achieving a metabolically mature model represents an important goal precisely because of the role that cellular metabolism plays in pathophysiological states already defined in the clinic but not yet achieved in in vitro studies.

## 5. Conclusions

Having a cellular model for studying cardiac pathophysiology is a very important target in both basic research and translational medicine. HiPSC-CMs provide a reliable and highly investigated model. However, one limitation of hiPSC-CMs is their metabolic immaturity. The fetal energy supply of 2D cultures makes this model unsuitable for studying cardiometabolic diseases, while 3D culture systems have emerged as superior models for studying both physiological and pathological cardiac conditions and for drug discovery and screening. For in vitro maturation protocols or disease modeling, using a combination of metabolic, structural, and functional markers provides a more accurate assessment of maturation state. Reliance on single parameters (e.g., mitochondrial morphology or one sarcomeric gene) may be misleading, as these features can be hierarchical and not always developed to the same extent.

In this scenario, several studies revealed the importance of a cellular model that could be used for the assessment of cardiotoxicity, especially concerning new pharmacological approaches and with a view to personalized medicine [68,80]. As comprehensively reviewed by Magdy et al., the current generation of hiPSC-CMs recapitulate some of the fundamental characteristics of the native cardiomyocyte; however, there are still important differences concerning functional and metabolic maturation that do not allow their definitive use in drug cardiotoxicity screening and personalized medicine [78].

**Figure 2:**
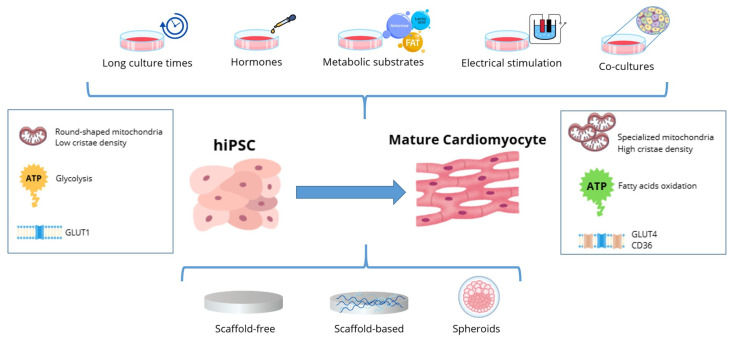
Schematic representation of several strategies used in 2D and 3D models to induce hiPSC-CMs’ metabolic maturation. Image created with Canva.com.

In summary, significant results have been achieved in both 2D and 3D monoculture studies as well as in more complex multiculture systems, although standardized and widely accepted protocols for hiPSC-CMs metabolic maturation are needed to develop a valuable cell model for studying various pathological conditions affecting the cardiac tissue/heart and to use them as a model to assess drug development, safety and toxicity screening (Figure 2).

## Abbreviations

The following abbreviations are used in this manuscript:

ES	Embryonic Stem
iPSCs	Inducible Pluripotent Stem Cells
hiPSC-CMs	Human Inducible Pluripotent Stem Cell-derived cardiomyocyte
T3	Triiodothyronine
CM	Cardiomyocyte
ATP	Adenosine Triphosphate
GLUT	Glucose Transporter
FFAs	Free Fatty Acids
cAMP-PKA	Cyclic Adenosine Monophosphate-Protein Kinase A
pAKT	Phospho Protein Kinase B
AS160	Akt Substrate 160
IGF-1	Insulin-like Growth Factor-1
TID	T3, IGF-1 and dexamethasone
CD36	Long Chain Fatty Acid Transporter
FABPpm	Plasma Membrane isoform of Fatty Acid Binding Protein
FATP	Fatty Acid Transport Protein
UCP	Uncoupling Protein
FASN	Fatty Acid Synthase
PDK	Pyruvate Dehydrogenase Kinase
hOCMs	Human Organotypic Cardiac Microtissues
